# Evaluating lowland coffee genotypes against leaf rust and wilt diseases in southwestern Ethiopia

**DOI:** 10.3389/fpls.2025.1560091

**Published:** 2025-07-16

**Authors:** Hailu Negesa, Gabisa Gidisa, Zenebe Wubshet, Desalegn Alemayehu, Kifle Belachew, Wakuma Merga, Lemi Beksisa, Dawit Merga, Mohammedsani Zakir

**Affiliations:** ^1^ Department of Plant Pathology, Jimma Agricultural Research Center, Ethiopian Institute of Agricultural Research, Jimma, Ethiopia; ^2^ Department of Coffee Breeding and Genetics, Jimma Agricultural Research Center, Ethiopian Institute of Agricultural Research, Jimma, Ethiopia; ^3^ Department of Coffee Breeding and Genetics, Teppi Agricultural Research Center Ethiopian Institute of Agricultural Research, Teppi, Ethiopia

**Keywords:** coffee genotypes, coffee leaf rust, coffee wilt disease, reaction, resistant

## Abstract

**Introduction:**

Coffee is one of the most economically essential agricultural commodities worldwide and in Ethiopia in particular. Despite its importance, it is constrained by different factors. Among these, coffee leaf rust, caused by *Hemileia vastatrix*, and wilt diseases, caused by *Gibberella xylarioides*, are major limiting factors of coffee production. However, Ethiopia has not yet reported a commercialized resistant variety for both of these diseases. Therefore, the present study was conducted to evaluate lowland coffee genotypes against coffee leaf rust and wilt diseases under field and greenhouse conditions, respectively.

**Methods:**

A field experiment was conducted across four locations (Agaro, Teppi, Bebeka, and Gelesha) from 2021 to 2023. A randomized complete block design with three replications was used. The experiment for wilt disease was conducted on seedlings using seedling stem-nicking inoculation techniques.

**Results:**

The results revealed a significant difference among the genotypes in reaction to leaf rust and wilt diseases. Among the tested genotypes, I-1, I-2, K-1, and K-2 consistently showed a highly resistant reaction to leaf rust across locations, whereas one genotype (EB-1) indicated a susceptible reaction across all locations. The highest mean leaf rust severity was recorded on EB-1 (27.1%), while the lowest severity was recorded on genotype I-2 (0.35%). Similarly, four genotypes (I-1, I-2, K-1, and K-2) showed moderate resistance to wilt disease and indicated an extended incubation period compared to the susceptible control (Geisha).

**Discussion:**

Analysis of genotype by environment (G×E) interaction indicated a highly significant interaction (P < 0.01). Among climate factors, relative humidity and maximum temperature showed a highly significant and positive correlation with coffee leaf rust. These resistant genotypes could be used by farmers as a component of integrated disease management in coffee leaf rust-prone areas of the country. In addition, end users must integrate these genotypes with other wilt management options. Overall, these genotypes can enhance the resilience of coffee production when combined with other management strategies for coffee leaf rust and wilt diseases across the lowland coffee production areas of Ethiopia.

## Introduction

Coffee is one of the most important cultivated crops around the world. It is the second most traded commodity after crude oil and employs more than 100 million people globally (Gray et al., 2013; Asegid, 2020). It creates significant trade relations between producing and consuming countries (Redden, 2022; Siles et al., 2022). It is an important exchange commodity that contributes significant national revenue for coffee-producing nations to various extents (Lemma and Megersa, 2021). Not only for producing countries, coffee has also been playing a pivotal role in the economies of consuming countries through different value chains. Thus, its significance extends beyond economic value; coffee also influences the livelihoods, international trade, environmental protection, and cultural values of the countries (Siles et al., 2022; Correia da Cruz et al., 2024; McCook, 2024). Globally, Ethiopia has been recognized as the birthplace of Arabica coffee (Girmay, 2024). Coffee is a primary export commodity that contributes more than 30% of foreign currency earnings in Ethiopia (Asfaw, 2023; Girmay, 2024). The total land area covered by coffee production in Ethiopia is estimated to exceed 700,000 ha (Daba et al., 2022). Uniquely, coffee has a lion’s share in the Ethiopian economy and the cultural well-being of the people (Worku, 2023); up to 5% of the country’s gross domestic product (GDP) is estimated to be covered by the coffee sector (Hailu and Wako, 2024). More than 20% of Ethiopian people depend on coffee production, particularly in rural areas, through employment and income opportunities (Adane, 2024). In addition to direct income, coffee plays a significant role in biodiversity conservation and ecological balance across the country (Legesse, 2020; Singh, 2022). However, despite its importance, coffee production is constrained by different biotic and abiotic factors. Among the constraints, coffee diseases—namely berry disease, wilt disease, leaf rust, thread blight, bacterial blight, and root rot—are significantly impacting coffee production and productivity in Ethiopia, which directly leads to the country’s economic repercussions (Belachew et al., 2015a, Belachew et al., 2015b; Demelash and Kifle, 2018).

Among the diseases, coffee leaf rust and wilt diseases are seriously affecting coffee production, especially in the lowland coffee-growing areas of Ethiopia (Chala et al., 2010; Daba et al., 2023). Globally, coffee leaf rust is the most important coffee disease and is estimated to cause up to 25% yield losses (Talhinhas et al., 2017; Daba et al., 2022). In Ethiopia, the incidence and severity of coffee leaf rust have been reported to be 86.7% and 55.5%, respectively (Belachew et al., 2020; Berihun and Alemu, 2022). In a similar way, wilt disease has also been an ongoing concern for Ethiopian coffee farmers for the past 70 years (Girma et al., 2010; Peck and Boa, 2024), and remains an alarming problem. Coffee wilt disease can lead to substantial yield losses and has been affecting the livelihoods of coffee farmers across the country (Flood, 2021; Mulatu et al., 2023); it is a critical challenge to the Ethiopian coffee sector. Nationally, the incidence and severity of wilt disease have been reported to be 28% and 5%, respectively (Wassie, 2019). Two decades ago, yield loss due to wilt disease was estimated at 3,360 tons annually (Oduor et al., 2003); while the current loss due to this disease can be estimated to have increased by several folds since reports indicated that within the last two decades, the incidence of wilt disease increased from 30% in 2003 to 42% in 2017, which implies that the impact of coffee wilt disease has increased over time (Peck and Boa, 2024).

In Ethiopia, studies on coffee leaf rust have mainly focused on the spatial distribution of the disease (Belachew et al., 2020; Daba et al., 2022), evaluation of bioagents against it (Zewdie et al., 2021; Colman et al., 2021; Ayalew et al., 2024), analysis of the pathogen’s genetic diversity (Daba et al., 2024), and the interaction of the disease with environmental conditions (Belachew et al., 2020; Zewdie et al., 2021). The status of coffee leaf rust has evolved significantly, becoming a major threat in regions previously considered less affected (Berihun and Alemu, 2022). The disease has been spreading from lowland to mid- and highland areas, with alarming increases in intensity over time (Belachew et al., 2020; Adem and Amin, 2021). Apart from Ethiopia, coffee leaf rust causes significant economic losses in more than 50 Arabica coffee-growing countries, drawing the attention of international communities for its management (Gichuru et al., 2021). However, in Ethiopia, limited efforts have been made to develop effective mitigation options. Among the control options, the use of resistant genotypes is often prioritized by researchers as a key component of an integrated management strategy that can be used in coffee cropping systems (Gichuru et al., 2021; Sera et al., 2022; Ferrucho et al., 2024). Nonetheless, very little effort has been made to evaluate the reaction of coffee genotypes to coffee leaf rust in Ethiopia. Coffee production across different environments is seriously constrained by diseases, with coffee leaf rust being one of the major threats (Belachew et al., 2020). The intensity of coffee diseases can vary across the agroecologies, which may challenge the stability of genotypes in their response to these diseases.

Moreover, genotype, environment, and their interaction can determine the yield and resistance performance of crops (Mohebodini et al., 2015). Thus, analysis of genotype × environment (G×E) interactions is crucial when the potential of genotypes varies across environments (De Leon et al., 2016). Indeed, identifying stable genotypes that exhibit minimal G×E interaction under significant environmental fluctuation is very important. Coffee production across different environments is seriously constrained by diseases, with coffee leaf rust being one of the major threats (Belachew et al., 2020). The intensity of coffee diseases can vary across agroecologies, posing a challenge to the stability of genotypes in their disease reactions. Therefore, G×E analysis is crucial for understanding genotype stability across different locations.

Regarding coffee wilt disease, despite extensive attempts to develop resistant coffee genotypes, there has been no commercially released wilt-resistant variety to date in Ethiopia. In addition, different cultural management options such as uprooting and burning infected trees, delaying replanting, avoiding wounds, disinfecting tools, using cover crops, applying mulch, and using bioagents have been recommended to manage wilt disease (Belachew et al., 2015a; Assefa et al., 2022; Mulatu et al., 2023). However, no resistant coffee genotype has been recommended to integrate with these cultural management options. Among the management options, using resistant varieties is the most straightforward, economical, harmless, and effective to reduce yield losses caused by coffee leaf rust and wilt diseases. Therefore, this study was initiated to evaluate the reaction of lowland coffee genotypes against coffee leaf rust and wilt diseases in southwestern Ethiopia.

## Materials and methods

### Description of the study areas

**Figure 1:**
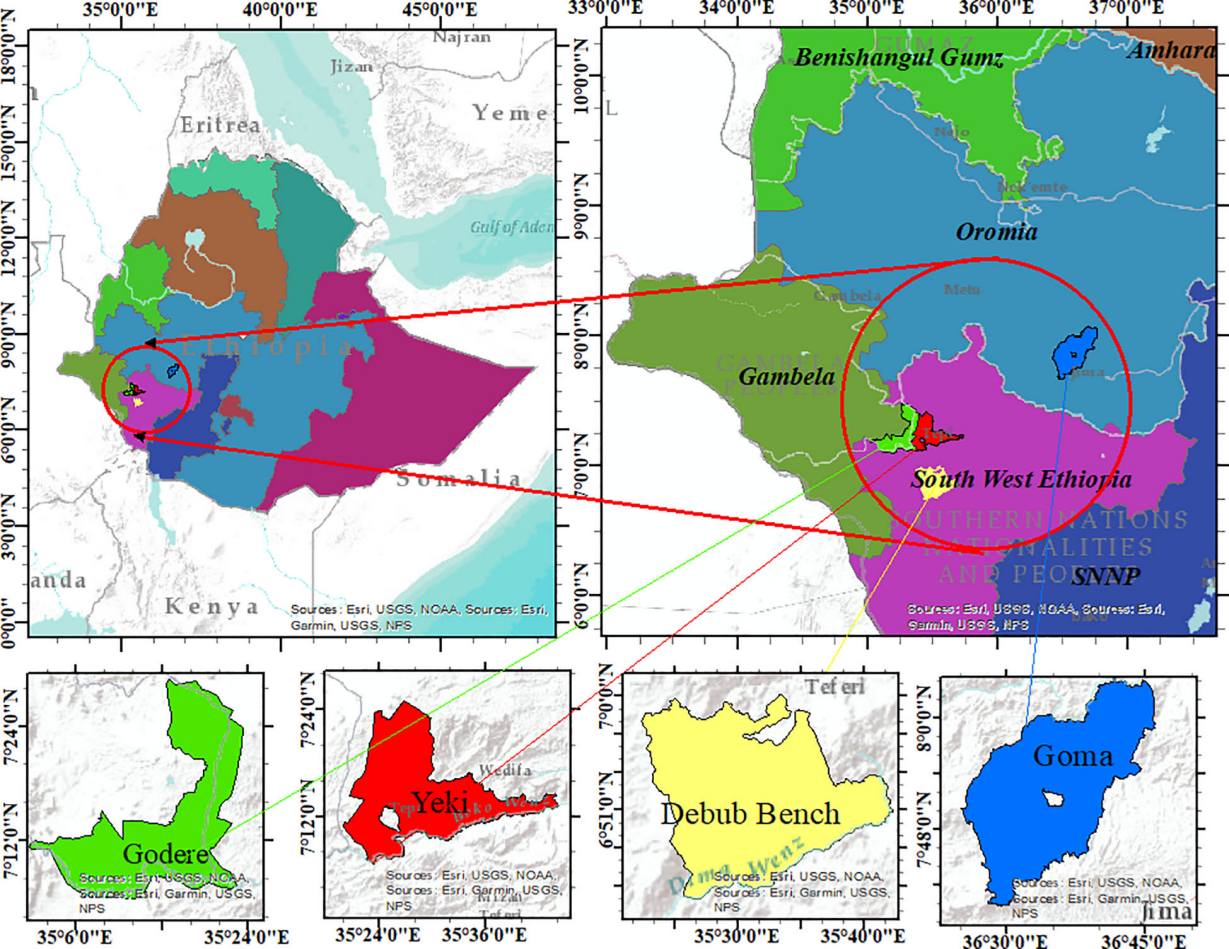
Map of the study areas.

The experiment was conducted in four different locations during the 2021 to 2023 cropping seasons. These locations were purposively selected to represent lowland and midland coffee-growing areas of Ethiopia, where coffee leaf rust and wilt diseases are major problems in coffee production (**Table 1**; **Figure 1**). Among the locations, the Gomma district is located in the Jimma zone, Oromia Regional State. The study was conducted at the Agaro Agricultural Research Subcenter, which is located in the Gomma district and was selected to represent midland coffee-growing areas (**Table 1**; **Figure 1**). This subcenter is located 397 km away from Addis Ababa (Abebe, 2005). The second experimental location was the Teppi Agricultural Research Center, located in the Yeki district, Sheka zone, in the southwestern Ethiopia People’s Regional State (Sora and Jibat Guji, 2023). The third experimental specific location was Bebeka, situated in the Debub Bench district, Bench Sheko zone, in the southwestern Ethiopia People’s Regional State, approximately 610 km from Addis Ababa (Abraham, 2020). The fourth experimental location was Gelesha, located in the Godere district, Majang zone, Gambela Regional State (Choudhary et al., 2021).

**Table 1:** Description of the study areas.

Districts	Specific locations	Altitude of specific locations (m)	Maximum average temperature	Minimum average temperature	Annual rainfall (mm)
Gomma	Agaro	1,630	28.4°C	12.4°C	1,616
Yeki	Teppi	1,200	29.7°C	15.5°C	1,559
Debub bench	Bebeka	1,368	35.6°C	17.2°C	1,742
Godere	Gelesha	1,000	33.1°C	13.2°C	2100 mm

### Experimental design and treatments for coffee leaf rust

Five coffee genotypes (EB-1, I-1, I-2, K-1, and K-2) were collected from an international coffee collection that was established and planted by the international coffee collection program at Bebeka, whereas coffee cultivars used as controls (7454 and Dessu for leaf rust as well as Feyate, Geisha, and 370 for wilt disease) were collected from Jimma agricultural research center (**Table 2**). The genotypes were selected based on their background for yield and disease resistance. The pedigree and mode of pollination of the genotypes and cultivars are pure lines and self-pollination, respectively. Subsequently, these genotypes were subjected to evaluation under different locations with two cultivar controls (7454 and Dessu), which are currently under production across the study areas. The study was laid out in a randomized complete block design (RCBD) across the locations.

**Table 2:** Description of coffee genotypes and varieties used for evaluation against leaf rust and wilt diseases in southwestern Ethiopia.

Coffee genotypes	Origin	Reaction to CLR	Reaction to wilt
I-1	International collection	Highly resistant	–
I-2	International collection	Highly resistant	–
K-1	International collection	Highly resistant	–
K-2	International collection	Highly resistant	–
EB-1	International collection	Susceptible	–
7454	Variety	Moderate resistant	–
Dessu	Variety	Resistant	–
Feyate	Variety	–	Resistant
Geisha	Variety	–	Susceptible
370	Genotype	–	Resistant

“–” reaction to the disease unknown.

### Experimental design and treatments for coffee wilt disease

Evaluation for wilt reaction was undertaken under greenhouse conditions at Jimma Agricultural Research Center (JARC) using five coffee genotypes (EB-1, I-1, I-2, K-1, and K-2), along with two positive controls (370 and Feyate) and one negative control (Geisha). The study was arranged in a completely randomized design (CRD) under greenhouse conditions with three replications (i.e., three pots per genotype).

### Seedling preparation for wilt experiment

Coffee seeds were collected from five coffee genotypes (EB-1, I-1, I-2, K-1, and K-2) and three controls (i.e., coffee cultivars 370 and Feyate as positive controls, and Geisha as a negative control). The seeds of each genotype and cultivar were soaked in sterile distilled water for 24 h after the parchment was removed to facilitate early germination. About 30 soaked seeds of each genotype were then sown into sterilized, moistened sandy soil in disinfected plastic pots. To maintain sufficient moisture for seedling growth, sterile water was routinely applied at 2-day intervals for 12 weeks (Belachew et al., 2015a). After germination, the number of seedlings was reduced to 20 per pot for inoculation.

### *Gibberella xylarioides* inoculum preparation

Samples of partially wilted stems were collected from the Gera area, and isolation was performed following the method described by Adugna et al. (2005). Synthetic nutrient agar (SNA) medium was used for isolation. After isolation, purification was carried out, and mass spore production was prepared using sterilized fresh coffee twigs. The twigs, collected from healthy trees (Geisha variety), were cut into small pieces (10 to 15 cm), and the bark was carefully scratched off. Subsequently, the twigs were sterilized in an autoclave. After sterilization, each twig was inoculated with three discs (5 mm) of *Gibberella* xylarioides and incubated for 7–10 days at 22°C ± 2°C (Adugna et al., 2005; Mulatu et al., 2023). The spore suspension was then prepared by thoroughly rinsing the twigs with sterilized water. The suspension was stirred with a magnetic stirrer and filtered through double layers of cheesecloth. Finally, the spore concentration was adjusted to 2 × 10^6^ conidia per milliliter using a hemocytometer.

### Inoculation of coffee seedlings

Inoculation of *Gibberella xylarioides* was performed using the stem-nicking (stem wounding) technique on 12-week-old coffee seedlings (Adugna and Huluka, 2000). A sterilized scalpel was dipped into the spore suspension and used to nick the stem about 2 cm above the soil level (Belachew et al., 2015a). After inoculation, the seedlings were kept in a controlled growth room with optimal relative humidity (> 95%) and temperature range (23°C–25°C) for 10 days to promote infection (Adugna et al., 2005; Belachew et al., 2015a). Subsequently, the seedlings were transferred to a greenhouse for continuous monitoring and data collection.

### Coffee leaf rust data collection

Leaf rust severity was assessed on three pairs of branches representing the upper, middle, and lower canopy layers of each coffee plant. For each genotype and cultivar, five representative trees were selected for data collection. On each branch, the number of leaves was counted and rated for leaf rust severity following the method of Julca et al. (2019). The average leaf rust severity per branch was calculated based on the proportion of rusted leaf area. Finally, the severity of leaf rust per tree was determined by segregating the values from each branch (Julca et al., 2019; Malau et al., 2021).

### Coffee wilt disease data collection

Data collection began a month postinoculation and continued for 6 months at 2-week intervals following the standard procedures of Tshilenge-Djim et al. (2011). The number of seedlings showing wilt symptoms was recorded every 14 days. In addition, the incubation period (number of days from inoculation to symptom appearance) was periodically recorded (Belachew et al., 2015a). Wilt disease percentage was calculated as the cumulative number of dead seedlings divided by the total number of seedlings (dead plus healthy) over 6 months (Wubshet et al., 2024).

### Statistical analysis

The severity of coffee leaf rust and wilt diseases was analyzed using SAS statistical software (SAS, 2016). The percentage data for leaf rust were transformed to angular values before performing statistical analysis. The least significant difference (LSD) test was used for treatment mean separation. Finally, the reaction class of coffee genotypes and cultivars was determined based on the percentages of leaf rust severity according to Malau and Sihotang (2023), with slight modification. Genotypes with severity percentages of 0, 0.1–5, 5.1–15, 15.1–25, 25.1–35, and > 35% were classified as immune, highly resistant, resistant, moderately resistant, susceptible, and highly susceptible, respectively. Parametric Pearson correlation analysis was used to assess the association of coffee leaf rust with climate variables during the data collection month (October for each year). The significance thresholds for the relationship between leaf rust (dependent variable) and climate variables (independent variables) were determined using the 95% and 99% prediction ellipses. Before conducting the combined analysis, the homogeneity of residual variance was tested using Bartlett’s homogeneity test. Coffee leaf rust data were analyzed using the PROC MIXED model (GLM) with the MIXED procedure of SAS (2016), corresponding to the statistical model: *Y_ij_ =* μ *+ G_i_ + R_j_ +* ϵ*_ij_* for an individual site, where *Y_ij_* is the plot value of each trait for the *i*th genotype and the *j*th replication, μ is the trial mean of the given trait, *G_i_* is the effect of genotype, *R_j_* is the effect of replications, and ϵ*_ij_* is the plot error. For the analysis across locations/environments, ANOVA was carried out for each trait using the SAS procedure, based on the statistical model *Y_ijk_* = μ + *E_k_* + *R(E)k*(*j*) +*G_i_* + *GE_ik_* + ϵ*_ijk_*. Here, *E_k_*, *R(E)k*(*j*), and *GE_ik_* represent the effects of locations/environments, the effect of replications nested within locations/environments, and the genotype–environment interaction, respectively. The statistical significance of these effects was determined using the *F*-test. Stability analysis was performed using GEA-R software version 4.0 (2016).

## Results

### Coffee leaf rust

The result indicated that there were significant differences among the genotypes in reaction to coffee leaf rust disease consistently across the locations (**Table 3**). Based on the mean disease severity percentage, four genotypes (I-1, I-2, K-1, and K-2) showed the lowest severity of leaf rust disease (**Table 3**), whereas the controls (7454 and Dessu) showed severity levels three times greater than the genotypes that showed a resistant reaction. Based on the categorization of reaction classes, the four genotypes (I-1, I-2, K-1, and K-2) exhibited highly resistant reactions to leaf rust disease (**Table 3**). These resistant genotypes have consistently demonstrated the capacity to resist coffee leaf rust across locations and seasons. On the contrary, the EB-1 genotype showed a susceptible response to leaf rust, whereas the controls, 7454, and Dessu exhibited moderately resistant and resistant responses, respectively (**Table 3**). The highest mean leaf rust severity was recorded on EB-1 (27.1%), while the lowest severity was recorded on the I-2 (0.35%) genotype. A wide range of leaf rust severity percentages was observed among the genotypes. In highly resistant genotypes, a leaf rust severity ranging from 0% to 3% was recorded, whereas 7% to 40% severity was observed in the susceptible genotype (EB-1).

**Table 3:** Severity percentage of coffee leaf rust and reaction class of coffee genotypes in southwestern Ethiopia, 2020 to 2022.

Genotypes	Agaro		Bebeka			Teppi			Gelesha			RC
	CLR		CLR			CLR			CLR			
	2021	2022	2020	2021	2022	2020	2021	2022	2020	2021	2022	
EB-1	36.3 (3.5) ^a^	36.7 (3.5) ^a^	7.0 (1.4) ^ab^	15.4 (2.3) ^a^	52.1 (4.2) ^a^	15.7 (2.3) ^a^	24.3 (2.9) ^a^	32.5 (3.3) ^a^	8.0 (1.6) ^ab^	39.6 (3.6) ^a^	30.4 (3.2) ^a^	S
I-1	3.4 (1.2) ^d^	0.6 (0.7) e	3.3 (1.0) ^ab^	0.0 (0.6) ^c^	0.0 (0.6) ^d^	0.0 (0.6) ^c^	0.0 (0.6) ^d^	0.0 (0.6) ^d^	3.7 (1.1) ^bc^	0.0 (0.6) ^c^	0.0 (0.6) ^b^	HR
I-2	3.6 (1.2) ^d^	0.2 (0.6) e	0.0 (0.6) ^b^	0.0 (0.6) ^c^	0.0 (0.6) ^d^	0.0 (0.6) ^c^	0.0 (0.6) ^d^	0.0 (0.6) ^cd^	0.0 (0.6) ^c^	0.0 (0.6) ^c^	0.0 (0.6) ^b^	HR
K-1	3.4 (1.2) ^d^	2.8 (1.1) ^d^	0.0 (0.6) ^b^	0.0 (0.6) ^c^	0.0 (0.6) ^d^	0.0 (0.6) ^c^	0.0 (0.6) ^d^	0.0 (0.6) ^d^	0.0 (0.6) ^c^	0.0 (0.6) ^c^	0.0 (0.6) ^b^	HR
K-2	6.1 (1.5) ^c^	0.2 (0.6) e	1.3 (0.8) ^b^	0.0 (0.6) ^c^	5.0 (1.4) ^c^	3.3 (1.0) ^bc^	5.0 (1.3) ^c^	10.4 (1.9) ^c^	1.3 (0.8) ^bc^	4.6 (1.1) ^c^	0.8 (0.7) ^b^	HR
Dessu	25.0 (2.9) ^b^	4.2 (1.3) ^c^	3.3 (1.1) ^ab^	5.0 (1.4) ^b^	25.4 (2.9) ^b^	5.3 (1.4) ^b^	14.3 (2.2) ^b^	21.7 (2.7) ^ab^	4.0 (1.3) ^ab^c	23.3 (2.8) ^b^	20.8 (2.6) ^a^	R
7454	26.3 (3.0) ^b^	9.6 (1.9) ^b^	11.3 (1.9) ^a^	12.1 (2.0) ^a^	39.2 (3.6) ^a^	15.3 (2.3) ^a^	18.4 (2.5) ^ab^	18.7 (2.5) ^bc^	11.7 (1.9) ^a^	25.4 (2.9) ^ab^	30.4 (3.2) ^a^	MR
LSD	0.2	0.16	0.9	0.5	0.6	0.7	0.5	0.7	0.8	0.8	0.7	
CV (%)	6.5	6.5	51.7	23.2	18.0	34.3	19.8	21.7	38.2	24.7	23.3	

The number in parentheses is the transformed value using arcsine. Mean values with the same letter within a column did not significantly differ at *p* < 0.05. *RC*, reaction class; *S*, susceptible; *HR*, highly resistant; *R*, resistant; *MR*, moderate resistant; *LSD*, least significance difference; *CV*, coefficient of variation; *CLR*, coffee leaf rust.

### Coffee wilt disease

The analysis revealed a significant difference among the genotypes in their reaction to wilt disease (**Table 4**). This demonstrates the presence of genetic variability among Arabica coffee genotypes in response to wilt disease, encouraging further investigation and providing insights for developing resistant coffee varieties. Among the tested genotypes, I-1, I-2, K-1, and K-2 exhibited moderate resistance to wilt disease under greenhouse conditions (**Table 4**). Furthermore, compared to the susceptible control, these genotypes displayed an extended incubation period. On the other hand, the resistant controls (370 and Feyate) performed significantly better than the genotypes exhibiting a moderate resistance to wilt disease. Among the tested genotypes, Feyate showed no wilt symptoms throughout the data collection period, indicating its strong resistance potential as a control genotype. In contrast, negative control (Geisha) exhibited the highest severity of the disease and the fastest incubation period compared to the other tested genotypes. In general, this finding confirmed the existence of variability in reaction to wilt disease, ranging from susceptible to highly resistant ranges. The EB-1 genotype was not tested due to failure to germinate; therefore, there were no results on its reaction to wilt disease.

**Table 4:** Severity of coffee wilt disease on genotypes under greenhouse conditions.

Genotypes	Disease severity (%)	IP	Remark
EB-1	NE	NE	
I-1	45.3 b	90	
I-2	38.9 b	110	
K-1	33.3b	95	
K-2	42.5b	100	
370	8.1c	165	Positive control
Feyate	0.0 c	0.0	Positive control
Geisha	97.2 a	67	Negative control
Mean	37.9	89	
CV (%)	17.7		
LSD	19.8		

NE, not evaluated for wilt disease, not tested due to germination loss of the seed; IP, incubation period.

## Discussion

### Coffee leaf rust

Coffee leaf rust is one of the main global challenges of coffee production. Therefore, one of the resilient mitigating ways is developing resistant coffee varieties. However, in this country, there has been a limitation of coffee leaf rust-resistant varieties to date. The current study identified coffee leaf rust-resistant genotypes across the locations. Similar to our result, Malau et al. (2021) reported coffee genotypes that were resistant to leaf rust across locations. Among the genotypes, I-1, I-2, K-1, and K-2 showed highly resistant reactions (**Table 3**). Concurrently, different studies identified Arabica coffee genotypes that exhibited a resistant reaction to coffee leaf rust (Belete et al., 2014; Malau et al., 2021; Aryal et al., 2022). In addition, previous findings identified coffee genotypes that showed moderate resistance to leaf rust (Gokavi et al., 2022), which agrees with the current result recorded for the 7454 genotype. In this study, genotypes such as EB-1, Dessu, and 7454 showed moderately resistant and resistant reactions in some of the study years, which could have been due to the age of the crop, as the lowest severity was recorded in the 2020 production year. Our finding matches that of Ehrenbergerova et al. (2018), who reported that coffee plant age had a significant positive effect on the intensity of coffee leaf rust. Stability analysis also indicated that the EB-1 genotype was not stable across the locations (**Figure 3**). This indicates the inconsistent performance of a genotype under different environmental conditions, which can significantly affect the resistance and other traits of the genotype (Posada et al., 2009). Even though the genotypes showed a consistent reaction across locations and seasons, some slight differences observed in the severity of leaf rust across locations and seasons might have occurred due to the variations in environmental conditions, agronomic practices, and coffee ages from season to season (Rocha et al., 2015; Aryal et al., 2022). Similarly, previous studies stated that the expression of leaf rust resistance can be influenced by environmental conditions, which affect the physiological activity of coffee plants, pathogenesis, and sporulation of the pathogen (Toniutti et al., 2017; Malau et al., 2024).

**Figure 2:**
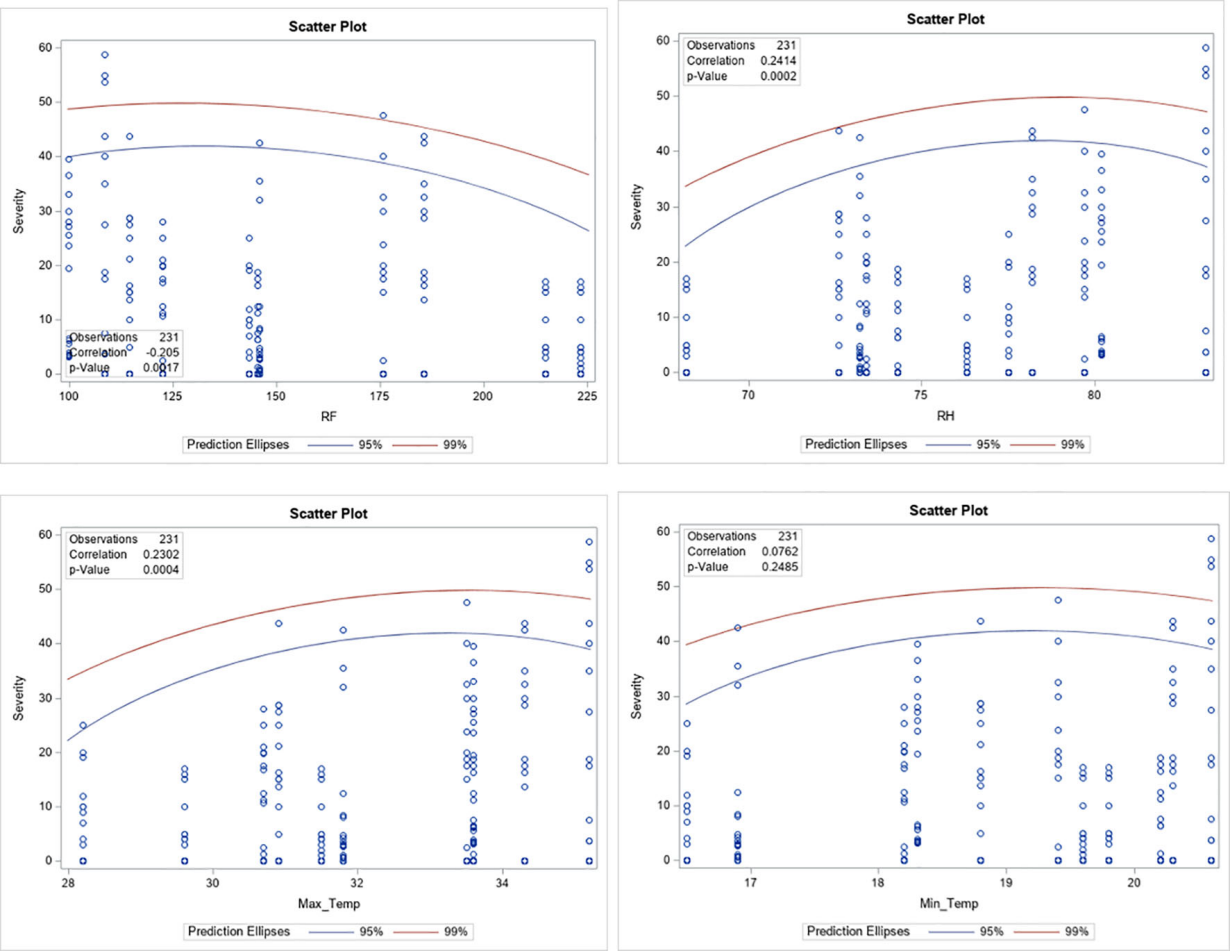
Correlation between coffee leaf rust severity and climatic factors. RH, relative humidity; RF, rainfall; Max_Temp, maximum temperature; Min_Temp, minimum temperature.

**Figure 3:**
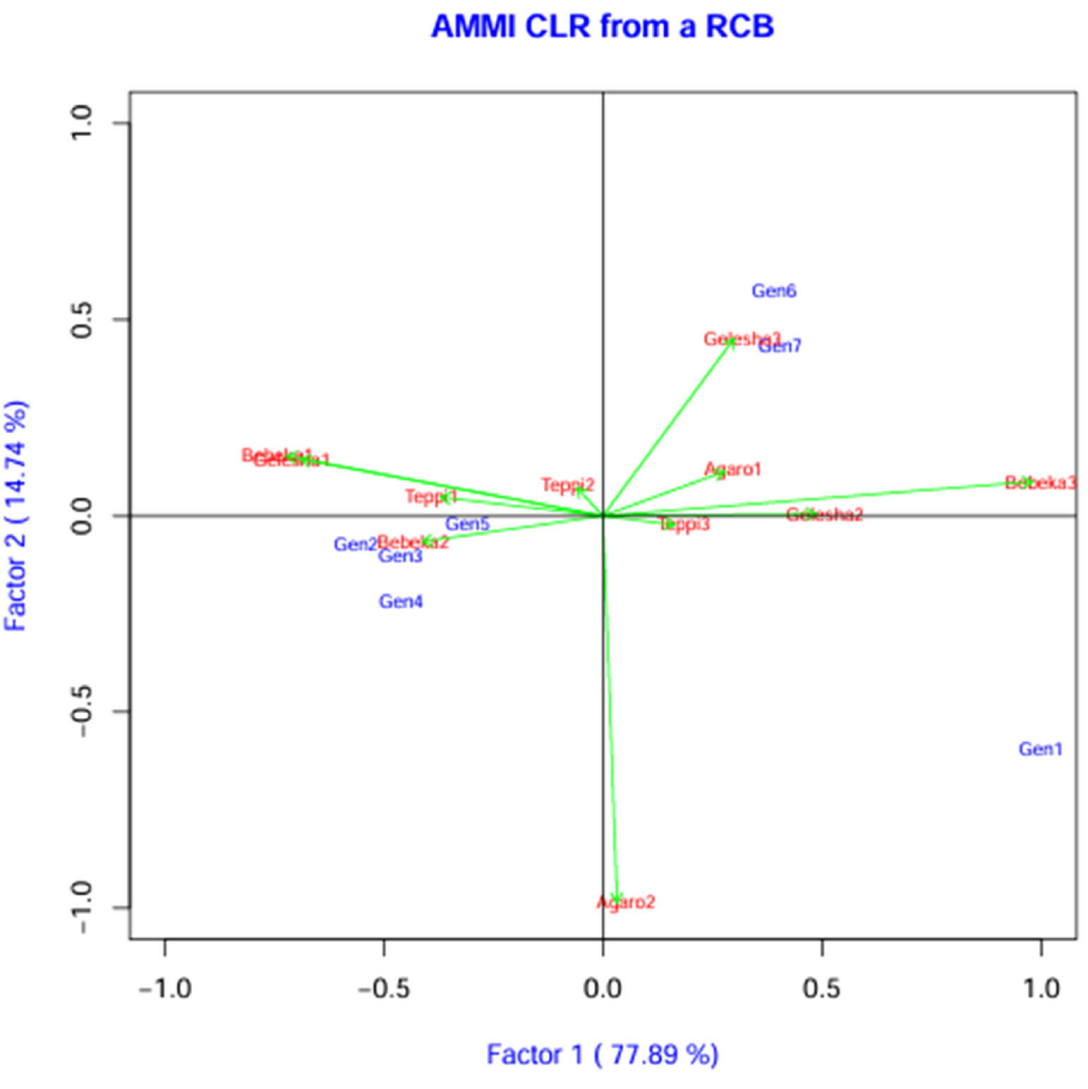
AMMI biplot of IPCA1 against coffee leaf rust severity percentages. Gen1, EB-1; Gen2, I-1; Gen3, I-2; Gen4, K-1; Gen5, K-2; Gen6, 7454; Gen7, Dessu.

Across the study locations, four genotypes (I-1, I-2, K-1, and K-2) consistently demonstrated a highly resistant reaction to leaf rust. Stability analysis also confirmed the stability of these genotypes across environments (**Figure 3**). Our findings align with previous studies, which reported that the response of coffee genotypes against leaf rust disease is mainly influenced by genetic resources and environmental conditions (Aryal et al., 2022; Mariz et al., 2025). Additionally, correlation analysis showed that rainfall (**Figure 2A**, **Table 5**) had a significantly negative correlation with the severity of coffee leaf rust. Meanwhile, relative humidity and maximum temperature (**Figures 2B, C**, **Table 5**) showed a highly significant and positive correlation with the severity of leaf rust. Among the climatic factors, no variable exhibited a strong correlation with the severity of leaf rust; this suggests that genetic resistance of the coffee plant, possible virulence of the pathogen, and microclimate conditions played significant roles in the slight variability of disease severity observed across the locations. Our findings of no strong correlation between climate variables and coffee leaf rust severity align with the previous study by Malau et al. (2024), which reported that genetic factors of the host and pathogen can significantly influence disease outcomes.

**Table 5:** Correlation between coffee leaf rust and climate variables of October during the 2020 to 2022 production years.

Variables	Max.T	Min.T	RF	RH	Sev
Max.T	1	0.63^**^	− 0.21^*^	0.64^**^	0.23^**^
Min.T	0.63^**^	1	0.29^**^	0.17^*^	0.08^ns^
RF	− 0.21^*^	0.29^**^	1	− 0.34^**^	− 0.21^*^
RH	0.64^**^	0.17^*^	− 0.34^**^	1	0.24^**^
Sev	0.23^**^	0.08^ns^	− 0.21^*^	0.24^**^	1

*Max.T*, maximum temperature; *Min.T*, minimum temperature; *RF*, rainfall; *RH*, relative humidity; *Sev*, leaf rust severity; *ns*, nonsignificant; ^*^significant; ^**^highly significant.

In general, a significant difference was observed among the coffee genotypes against leaf rust, among which four genotypes were highly resistant. These genotypes consistently maintain their leaf rust resistance across the locations under different environmental conditions. Therefore, these coffee genotypes have the potential to enhance coffee production and productivity across the lowland coffee-growing areas of the country. In addition to being released as varieties, they can also serve as sources of genes for developing resistant varieties in future breeding programs. Thus, it is crucial to adopt these resistant genotypes as one of the main mitigation options and also to consider and manage environmental conditions to minimize the damage caused by coffee leaf rust. In addition, understanding the evolving nature of the pathogen requires attention to effectively and sustainably address the issue of coffee leaf rust.

### Genotype-by-environment interaction

Phenotypic expression of one genotype against coffee leaf rust that performs better in one environment might be less effective in another environment, and such background can be determined by analysis of environment-by-genotype interaction. In the present result, genotypes showed highly significant interaction, whereas the environment did not significantly interact with the severity of leaf rust disease. This indicates that genetic potential can play a more crucial role in determining leaf rust severity than environmental conditions. Previous findings also stated that genotypes exhibited highly significant interactions with coffee leaf rust severity percentages, whereas the environment did not show a significant interaction with the severity of coffee leaf rust disease. Therefore, the differences in disease severity were primarily determined by the genetic makeup of the genotypes rather than environmental factors, which strongly indicates the importance of genotype in managing coffee leaf rust disease (Rodrigues et al., 2015; Malau, 2020).

### Stability analysis

#### Additive main effects and multiplicative interaction

The additive main effects and multiplicative interaction (AMMI) analysis of variance indicated highly significant differences (*p* < 0.01) for environments, genotypes, and their interaction. Moreover, Gollob’s test revealed that the first two IPCAs were highly significant (*p* < 0.01), indicating that the total information contained in the genotype-by-environment interaction can be explained using these IPCAs (**Table 6**). The cumulative value of PC1 and PC2 was 92.63, and the pattern of GEI by PC1 against PC2 is generally informative, while the rest of the PCs were captured as noise (**Table 7**). Stability analysis depicted the large sum of squares and a highly significant mean square of genotype, which showed that the genotypes were very diverse in their reaction to leaf rust. This indicated that the influence of genotypes was greater than that of the environment on the severity of coffee leaf rust. This suggests that coffee genetic resources can play a critical role in determining the reaction of genotypes to coffee leaf rust, and managing coffee leaf rust may need to focus on selecting resilient genotypes across locations. In contrast to this finding, Malau et al. (2021) reported that the largest variation was due to the environment, while such a contrast might have occurred due to the variations in materials used for the experiment.

**Table 6:** Combined ANOVA for coffee leaf rust sum of squares of seven genotypes tested across 11 environments.

Source	*df*	SS	MS	*F*-value	Pr > *F*
Environment (E)	10	4,679.9	467.9	19.70	< 0.0001
Rep (environment)	22	841.6	38.3	1.61	0.0530
Genotype(G)	6	23,429.6	3,904.9	164.34	< 0.0001
GEIs	60	7,324.6	122.1	5.14	< 0.0001
Error	132	3,136.5	23.8		
Total	230	39,370.3			

df, degree of freedom; SS, sum of squares; MS, mean square; GEIs, genotype-by-environment interaction.

**Table 7:** Combined analysis of variance over environments (locations).

Variances	*df*	SS	Explained	% Cum	MS	*F*-value	*p*-value
Env	10	4,642.0	13.1	13.1	464.2	17.9	0
Gen	6	23,420.7	66.2	79.3	3,903.4	151.1	0
Env*Gen	60	7,329.5	20.7	100	122.2	4.7	0
PC1	15	5,700.6	77.8	77.9	380.0	15.4	0
PC2	13	1,078.8	14.7	92.6	82.9	3.4	0.00015
Residuals	154	3,978.1	0.0	0.0	25.8	NA	NA

df, degree of freedom; SS, sum of squares; MS, mean square; Env, environment; Gen, genotype; PC1, principal component one; PC2, principal component two; NA, not available.

**Figure 4:**
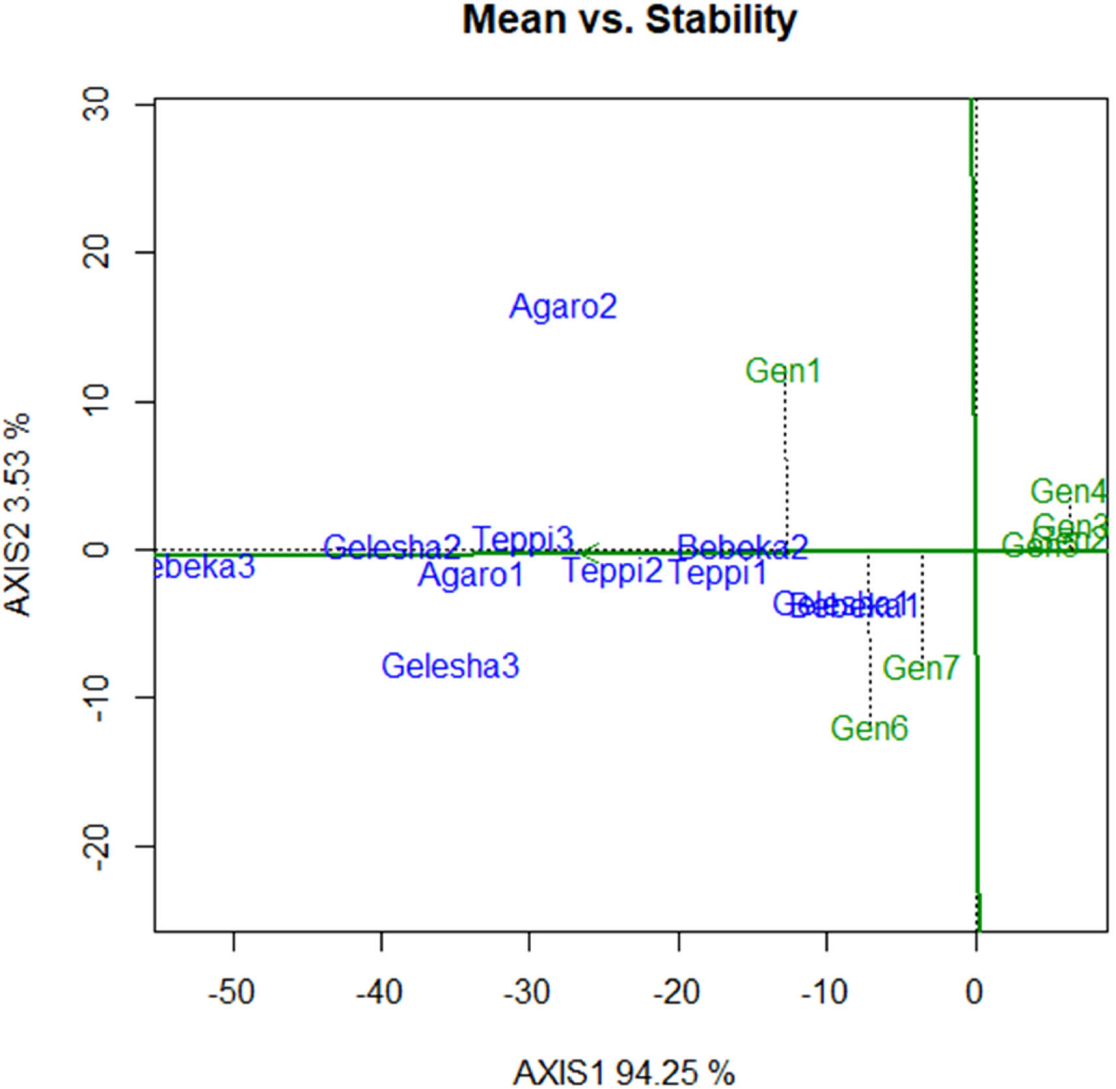
Mean versus stability biplot analysis using leaf rust severity percentages.

The mean versus stability biplot clearly showed the mean performance and stability of the genotypes (**Figure 4**). The genotypes along the average environment coordinate axis, with an arrow indicating the highest value, represent their mean performance across all environments (**Figure 4**). The average environment coordinate ordinate separates genotypes with below-average means from those with above-average means. In this regard, candidate genotypes I-1, I-2, K-1, and K-2 showed lower mean reactions to coffee leaf rust. In the AMMI 1 biplot model, the IPCA 1 scores of genotypes and environments were plotted against their respective means (**Table 8**; **Figure 3**). The IPCA scores for both genotypes and environments were plotted against the coffee leaf rust reaction for genotypes and environments. Genotypes or environments on the right side of the midpoint of the axis have lower reactions to coffee leaf rust than those on the left side (**Figure 3**). Accordingly, genotypes I-1, I-2, K-1, and K-2 are plotted on the right side of the main axis and exhibited lower coffee leaf rust severity than the genotypes plotted on the left side of the main axis (**Figure 3**). Therefore, this implies that the four coffee genotypes described above were coffee leaf rust-resistant genotypes (**Figure 3**).

**Table 8:** List of the environments and their descriptions used to analyze GXE interaction.

Environment	Description	Environment	Description
Teppi-1	2020/2021	Gelesha-1	2020/2021
Teppi-2	2021/2022	Gelesha-2	2021/2022
Teppi-3	2022/2023	Gelesha-3	2022/2023
Bebeka-1	2020/2021	Agaro-1	2021/2022
Bebeka-2	2021/2022	Agaro-2	2022/2023
Bebeka-3	2022/2023		

#### Coffee wilt disease

Similar to coffee leaf rust, coffee wilt disease has significantly impacted coffee production and productivity in Ethiopia. The disease remains economically important and, despite the availability of effective cultural management practices, varietal development addressing this disease is still lacking. Our results showed significant differences among genotypes in their reaction to wilt disease, consistent with previous studies reporting relatively resistant coffee genotypes (Getaneh et al., 2021; Olal et al., 2018). This highlights the presence of genetically determined variability among Arabica coffee genotypes in their response to wilt disease. In this study, some genotypes exhibited a longer incubation period compared to the susceptible control; however, these were significantly shorter than those observed in the resistant controls (370 and Feyate). In line with this result, Wubshet et al. (2024) recently reported accessions showing resistant and moderately resistant reactions to wilt disease under greenhouse conditions. Additionally, numerous previous studies have identified resistant and moderately resistant coffee genotypes under greenhouse conditions (Demelash and Kifle, 2015; Belachew et al., 2015a; Demelash Teferi et al., 2018). However, none of these previously reported genotypes demonstrated promising resistance under field conditions (personal observation). Our result was consistent with these previous findings. This implies that, when using these genotypes, integration with other cultural wilt management practices should be carefully considered. In short, this study also confirmed the existence of variability in the response to wilt disease among Arabica coffee genotypes, as previously reported by various studies (Girma et al., 2010; Demelash Teferi et al., 2018; Getaneh et al., 2021; Wubshet et al., 2024). Among the genotypes, Eb1 was not tested due to failure to germinate; therefore, no data were obtained on its reaction to wilt disease.

## Conclusion

Ethiopia is the birthplace of Arabica coffee and the center of its diversity. Similarly, the country has a highly diversified agro-ecology suitable for coffee production, which faces various biotic and abiotic production bottlenecks. Among these constraints, coffee diseases are highly economical; indeed, the coffee research strategy strongly emphasizes the development of disease-resistant genotypes for each of the growing areas. Nevertheless, despite extensive efforts to develop resistant coffee genotypes against wilt and leaf rust disease, no disease-resistant variety has been developed to date. Obviously, growing resistant coffee varieties has always been considered the most sustainable and affordable management option against coffee diseases. Thus, our study aimed to evaluate coffee genotypes against leaf rust and wilt disease. The study has identified coffee genotypes that showed resistant reactions to coffee leaf rust under field conditions and moderately resistant to wilt disease under greenhouse conditions. This implies the possibility of developing resistant coffee varieties through selection and hybrid development against these diseases. From the current finding, three coffee genotypes have been nationally released for leaf rust-prone areas but not for wilt disease, as these genotypes have not been evaluated under field conditions against wilt disease. Therefore, farmers must use them wisely by integrating recommended coffee wilt disease management options. Overall, the study identified highly resistant genotypes against leaf rust and recommended for these disease-prone areas. In addition, climate change is currently causing an increase in coffee disease epidemics across coffee-producing ecologies of Ethiopia. Thus, future research should focus on developing disease-resistant varieties by evaluating collections and hybrids across diverse ecologies. In addition, the identification and documentation of the resistance mechanisms in these genotypes should be studied to support future breeding programs.

## Data Availability

The original contributions presented in the study are included in the article/supplementary material. Further inquiries can be directed to the corresponding author.
